# Supplementary material for: Early unfractionated heparin treatment in patients with STEMI – trial design and rationale
Source: PLoS One. 2024 May 9;19(5):e0303376. doi: 10.1371/journal.pone.0303376 (PMC11081261; doi:10.1371/journal.pone.0303376)
Supplement: S1 Checklist — (DOC) [file pone.0303376.s001.doc]

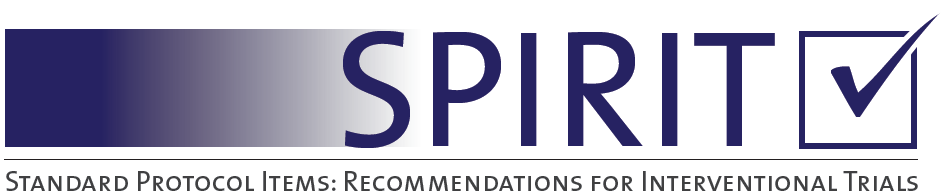


SPIRIT 2013 Checklist: Recommended items to address in a clinical trial protocol and related documents*

| Section/item | ItemNo | Description |
| --- | --- | --- |
| **Administrative information** | | |
| Title | 1 | Early unfractionated heparin treatment in patients with STEMI – trial design and rationale |
| Trial registration | 2a | [www.clinicaltrials.gov](http://www.clinicaltrials.gov/) : NCT05247424 |
|  | 2b | Data category: Information Primary registry and trial identifying number:  [www.clinicaltrials.gov](http://www.clinicaltrials.gov/) : NCT05247424  Date of registration in primary registry\|18 February 2022 Secondary identifying numbers\|/ Source(s) of monetary or material support\|University Medical Center Ljubljana, Slovenia  Primary sponsor\|University Medical Center Ljubljana, Slovenia Secondary sponsor(s)\|/  Contact for public queries\|Misa Fister, MD, PhD misa.fister@kclj.si  Contact for scientific queries\| Misa Fister, MD, PhD misa.fister@kclj.si  Public title\| Early unfractionated heparin treatment in patients with STEMI – trial design and rationale  Scientific title\| Early unfractionated heparin treatment in patients with STEMI – trial design and rationale  Countries of recruitment\| Slovenia Health condition(s) or problem(s) studied\| ST elevation myocardial infarction  Intervention(s)\| heparin or nothing  Key inclusion and exclusion criteria\| Inclusion criteria: adult patients with ST-elevation acute myocardial infarction, chest pain for less than 6 hours  Exclusion criteria: cardiac arrest without regaining consciousness, chest pain for more than 6 hours, hemodynamic impairment (cardiogenic shock), pregnant women  Study type\|Interventional~~Allocation: randomized~~Intervention model: parallel assignment~~Masking: blind (outcomes assessor)~~Primary purpose: spontaneous reperfusion before coronary angiography  Date of first enrolment\| March 2022 Target sample size\| 600 Recruitment status\| Recruiting Primary outcome(s)\| TIMI flow grade 2 or 3 on diagnostic coronary angiography  Key secondary outcomes\| final TIMI flow grades 2 and 3, highest Troponin I value, troponin I value 24 hours after PPCI, ST-segment resolution in a single lead with maximum baseline ST-segment elevation, progression to cardiogenic shock, 30-day mortality  frame: 3 years |
| Protocol version | 3 | January 2022; 1^st^ version |
| Funding | 4 | University Medical Center Ljubljana |
| Roles and responsibilities | 5a | Misa Fister, MD, PhD, University Medical Center Ljubljana and University of Ljubljana, Medical Faculty: design of protocol  Tomaz Goslar, MD, PhD, University Medical Center Ljubljana and University of Ljubljana, Medical Faculty: design of protocol  Prof. Marko Noc, MD, PhD, University Medical Center Ljubljana and University of Ljubljana, Medical Faculty: revision of protocol |
|  | 5b | / |
|  | 5c | Role of study sponsor and funders, if any, in study design; collection, management, analysis, and interpretation of data; writing of the report; and the decision to submit the report for publication, including whether they will have ultimate authority over any of these activities: / |
|  | 5d | Composition, roles, and responsibilities of the coordinating centre, steering committee, endpoint adjudication committee, data management team, and other individuals or groups overseeing the trial, if applicable (see Item 21a for data monitoring committee); / |
| Introduction |  |  |
| Background and rationale | 6a | Ischemic heart disease is the leading cause of death worldwide, accounting for 20% of all deaths in Europe. Mortality at 1 year in STEMI patients treated with coronary angiography is reported at approximately 10%. Immediate PPCI and opening of occluded coronary artery is the most important intervention that reduces STEMI-related mortality and morbidity. Guidelines for the management of patients with STEMI only recommend unfractionated heparin (UFH) at the time of PPCI, and the data on early treatment with UFH at the time of diagnosis are scarce.[1,2] Contrary to the international guidelines, our center has been using UFH at the dose of 70-100 IE/kg as a pretreatment immediately once the diagnosis of STEMI and the decision for PPCI was made.  In the proposed trial, we intend to investigate effectiveness and safety of early UFH administration compared to late administration at the time of coronary intervention. |
|  | 6b | In the recent COOL AMI EU Pivotal trial and the COOL AMI EU Pilot, the multicenter, prospective randomized controlled trials, we noticed a higher proportion of spontaneous reperfusions of culprit artery in our patients, receiving early UFH in the field when compared to other centers without early UFH treatment. |
| Objectives | 7 | The study will determine if early administration of UFH in the field at the time of established diagnosis and decision for PPCI is superior to the recommended application at the time of PPCI. |
| Trial design | 8 | Early unfractionated heparin treatment in patients with STEMI is a single-center, open-label, randomized controlled trial. |
| Methods: Participants, interventions, and outcomes | | |
| Study setting | 9 | Study population will consist of 600 STEMI patients referred for PPCI at University Medical Centre of Ljubljana, Slovenia. |
| Eligibility criteria | 10 | Inclusion criteria are based on standard definition of STEMI (at least two contiguous leads with ST-segment elevation ≥ 2.5 mm in men < 40  years, ≥2 mm in men ≥ 40  years, or  ≥ 1.5  mm in women in leads V_2_–V_3_ and/or ≥ 1 mm in the other leads in the absence of left ventricular hypertrophy or left bundle branch block)[1], with additional limitation for duration of symptoms.  Inclusion criteria are:   - Adult patients with ST-elevation acute myocardial infarction - Chest pain for less than 6 hours   Exclusion criteria are:   - Cardiac arrest without regaining consciousness - Chest pain for more than 6 hours - Hemodynamic impairment (cardiogenic shock) - Pregnant women |
| Interventions | 11a | Patient randomization is performed directly after establishing diagnosis of STEMI and decision for PPCI has been made. Randomization is in a 1:1 ratio to:   1. Immediate application of UFH intravenously at a dose of 70-100 units/kg on top of standard treatment. Additional UFH is added after diagnostic coronary angiography according to activated clotting time (ACT) when PPCI is performed. 2. Standard treatment and application of intravenous UFH only after diagnostic coronary angiography at a recommended dose of 70-100 units/kg when PPCI is performed.   When the ER doctor calls the intensivist about an eligible STEMI patient, randomization is performed using random permutated blocks via secure online randomization service ([www.sealedenvelope.com](http://www.sealedenvelope.com/)). The website can be opened on a smartphone or a computer. After entering the password for the study, the randomizing physician's email is entered, followed by the unique patient ID. The inclusion and exclusion criteria are then checked and the “Randomize" field is clicked. The result of the randomization to group A – heparin on the field - or B – no heparin - is displayed on the screen and sent to the randomizing physician's email. The doctor performing the randomization then informs the ER doctor about the therapy and dosage. |
|  | 11b | There is only one bolus of heparin in the field, so cross over or any other modification is not possible. |
|  | 11c | Intervention protocol only consists of randomising the patient and either giving the bolus of heparin or not. The doctor that does the randomisation informs the doctor in the field to either give the heparin or not. |
|  | 11d | The standard care of STEMI patient is given as per current guidelines. |
| Outcomes | 12 | Primary outcome  The primary efficacy endpoint of the study is TIMI[5] flow grade 2 or 3 on diagnostic coronary angiography.  Secondary outcomes  The secondary endpoints aim to assess infarct size, effectiveness of reperfusion, hemodynamic deterioration and survival.[6–8] Cardiogenic shock will be defined clinically (systolic blood pressure of less than 90 mmHg for longer than 30 minutes or the use of catecholamine therapy to maintain systolic pressure of at least 90 mmHg.[8]  Secondary endpoints are:   - Final TIMI flow grades 2 and 3 - Highest Troponin I value - Troponin I value 24 hours after PPCI - ST-segment resolution in a single lead with maximum baseline ST-segment elevation - Progression to cardiogenic shock - 30-day mortality |
| Participant timeline | 13 | 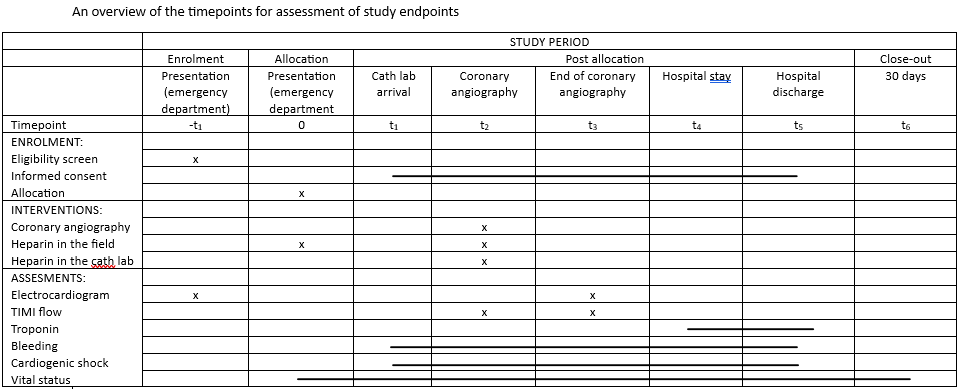 |
| Sample size | 14 | Several published observational studies have investigated pretreatment with UFH [10–13]. The reported difference in rates of spontaneous reperfusion varies and depends on the time between UFH administration and PPCI.  In a propensity-matched study of 552 matched patients, Bloom et al. reported a significantly lower proportion of patients with a TIMI 0 or 1 flow in the IRA in those who received UFH in fixed bolus doses of 4000 units and 1000 units at hourly intervals during transport prehospital (66% vs. 76%, p<0.001) compared with those who did not [11]. Investigators in the observational substudy of the TASTE trial reported a lower incidence of TIMI 0 or 1 in patients who received an average of 5000 units of UFH pre-hospital or in the emergency department (73.1% vs. 80.9%, p<0.001) compared to those who did not [12]. Another observational study by Giralt et al. also showed that pretreatment with UFH in a fixed intravenous dose resulted in a lower rate of TIMI 0 or 1 (69.7% vs. 78.8%, p<0.001) compared to the post-treatment group. In addition, the time-dependent effect of UFH administration was evident with higher rates of spontaneous reperfusion (TIMI 2 or 3) with shorter duration of administration from symptom onset [13].  The sample size calculation for this study is based on the historical baseline rate of TIMI 0 or 1 in STEMI patients arriving at our clinical center and the estimated benefit of prehospital UFH administration from the previously mentioned studies. The historical baseline rate of TIMI 0 or 1 flow at the University Medical Centre of Ljubljana is 58.5% with UFH pretreatment. We designed the study to detect a difference of 11.5% in the experimental group, which corresponds to 70% of patients with TIMI 0 or 1. To achieve a power of 80% for detecting this difference at a significance level of 5%, a total of 538 patients are required. In addition, we considered a dropout rate of 2.5%, which increases the total sample size required to 598. Sample size was calculated using the Sealed Envelope Ltd. 2012 Power Calculator for Binary Outcome Superiority Trial Web Application (https://www.sealedenvelope.com/power/binary-superiority/). |
| Recruitment | 15 | The doctors that recruit the patients are all members of the same department. They are reminded of the study periodically during staff meetings. |
| **Methods: Assignment of interventions (for controlled trials)** | | |
| Allocation: |  |  |
| Sequence generation | 16a | Patient randomization is performed directly after establishing diagnosis of STEMI and decision for PPCI has been made. Randomization is in a 1:1 ratio to:   1. Immediate application of UFH intravenously at a dose of 70-100 units/kg on top of standard treatment. Additional UFH is added after diagnostic coronary angiography according to activated clotting time (ACT) when PPCI is performed. 2. Standard treatment and application of intravenous UFH only after diagnostic coronary angiography at a recommended dose of 70-100 units/kg when PPCI is performed.   When the ER doctor calls the intensivist about an eligible STEMI patient, randomization is performed using random permutated blocks via secure online randomization service ([www.sealedenvelope.com](http://www.sealedenvelope.com/)). The website can be opened on a smartphone or a computer. After entering the password for the study, the randomizing physician's email is entered, followed by the unique patient ID. The inclusion and exclusion criteria are then checked and the “Randomize" field is clicked. The result of the randomization to group A – heparin on the field - or B – no heparin - is displayed on the screen and sent to the randomizing physician's email. The doctor performing the randomization then informs the ER doctor about the therapy and dosage. |
| Allocation concealment mechanism | 16b | This is described under 16a. |
| Implementation | 16c | The allocation and randomisation is done by intensivist doctor on call as described above. The emergency physician then either applies heparin or not to the patient according to the randomization group. |
| Blinding (masking) | 17a | The interventional cardiologists that afterwards analyses the coronary angiograms is blinded about the randomization group. The nurses that collect the data for the safety endopoint are also blinded about the randomization group. |
|  | 17b | The staff performing coronary angiography are not blinded for the allocation of the patient. The experienced cardiologist who subsequently evaluates TIMI flow is blinded, which does not influence the treatment of the patient. |
| **Methods: Data collection, management, and analysis** | | |
| Data collection methods | 18a | The ECRF for the study is available as a supplement to the paper. The data collection is performed by the allocating doctor and the doctors performing the study. The collected data are times of interventions, troponin levels, TIMI flow and bleeding complications. |
|  | 18b | The follow up is done via computer system in the hospital, and does not need the participation of the patient. After the patient signs informed consent, their involvement is no longer needed. The 30 day survival is checked via national health insurance validity. |
| Data management | 19 | The informed consents are stored in folders at the department in a locked room. The data for the study are entered into an online program RedCap. They are also stored in a separate folder in a computer. All data are only accessible by passwords. |
| Statistical methods | 20a,b,c | All data will be analyzed according to the intention-to-treat principle. Fisher’s exact test will be used to compare TIMI 2 or 3 flow rates in both arms. An odds ratio with a 95% confidence interval will be calculated as an estimator of early UFH administration. |
|  |  | For all secondary endpoints, the effect of early UFH administration with a corresponding 95% confidence interval will be estimated. |
| **Methods: Monitoring** | | |
| Data monitoring | 21a | Data monitoring committee will not be used. The study is simple, and the only difference in the study groups is the timing of heparin. We have used this practice in our center for many years without noticing any harm, and the same was seen in several retrospective studies. |
|  | 21b | There will not be any interim analysis of the study. |
| Harms | 22 | The harm of heparin would be bleeding, especially in the case of wrongful diagnosis (for instance aortic dissection or intracerebral bleeding with ECG changes mimicking STEMI). A UFH reversal is quickly available with protamine sulphate if needed.  We are collecting bleeding complications in our patients. However, due to a short action of heparin, the bleeding complications expected are not significant. |
| Auditing | 23 | There will not be any auditing in the study process. |
| Ethics and dissemination | | |
| Research ethics approval | 24 | The study was approved by the Ethics Committee of Republic of Slovenia (0120-591/2021/3) on January 20^th^ 2022. Since pretreatment with UFH is a standard of care in Slovenia and due to the nature of the emergency situation in the case of STEMI, Ethics Committee granted us a waiver to obtain informed consent at the time of randomization. Informed consent will be obtained at the time of PPCI or later during hospitalization, depending on mental condition of the patient. In case of death, Ethics Committee granted approval of data utilization without patient consent. |
| Protocol amendments | 25 | There will not be any protocol amendments to the study. |
| Consent or assent | 26a | This has been described under item 24. |
|  | 26b | This has been described under item 24. |
| Confidentiality | 27 | The ECRF is filled in by the intensivist on call. The forms are either paper or electronic, and stored in the locked hospital room of study investigators. The online program used for data entry is username and password protected. |
| Declaration of interests | 28 | There are no financial or competing interests of study investigators. |
| Access to data | 29 | All of the doctors that allocate the patients for the study have a separate username and password for data entry. Any unauthorised accesses are trackable by the program. |
| Ancillary and post-trial care | 30 | There are no expected harms of the trial. |
| Dissemination policy | 31a | The patients included in the study get an email of principal investigator of the study for any inquiries. After the results of the study will be analysed, the authors will publicate the manuscript in one of the medical journals listed in PubMed. |
|  | 31b | Proffesional writers will not be used. |
|  | 31c | The CRF, protocol, and data analysis will be fully available upon request. |
| Appendices |  |  |
| Informed consent materials | 32 | The consent form is a part of supplemental material of the manuscript. |

*It is strongly recommended that this checklist be read in conjunction with the SPIRIT 2013 Explanation & Elaboration for important clarification on the items. Amendments to the protocol should be tracked and dated. The SPIRIT checklist is copyrighted by the SPIRIT Group under the Creative Commons “[Attribution-NonCommercial-NoDerivs 3.0 Unported](http://www.creativecommons.org/licenses/by-nc-nd/3.0/)” license.
