# Supplementary material for: Early unfractionated heparin treatment in patients with STEMI – trial design and rationale
Source: PLoS One. 2024 May 9;19(5):e0303376. doi: 10.1371/journal.pone.0303376 (PMC11081261; doi:10.1371/journal.pone.0303376)
Supplement: Attachment — Submitted filename: Reviewer comments.docx [file pone.0303376.s002.docx]

The authors have prepared the study protocol on “Early unfractionated heparin treatment in patients with STEMI – trial design and rationale”. This study will provide important evidence. The protocol in general has been well written. I have made the following comments to get more clarity of the study.
1. Please elaborate the randomization process a bit more.
2. Please state how investigators would ensure blinding of assessors.
3. Please mention whether the time period from the admission to the start of intervention at the catheter laboratory is captured as a variable since it could be a confounder for the outcome of the study.
